# The Mych Gene Is Required for Neural Crest Survival during Zebrafish Development

**DOI:** 10.1371/journal.pone.0002029

**Published:** 2008-04-30

**Authors:** Sung-Kook Hong, Michael Tsang, Igor B. Dawid

**Affiliations:** 1 Laboratory of Molecular Genetics, National Institute of Child Health and Human Development, National Institutes of Health, Bethesda, Maryland, United States of America; 2 Department of Microbiology and Molecular Genetics, University of Pittsburgh, School of Medicine, Pittsburgh, Pennsylvania, United States of America; National Institutes of Health, United States of America

## Abstract

**Background:**

Among Myc family genes, *c-Myc* is known to have a role in neural crest specification in *Xenopus* and in craniofacial development in the mouse. There is no information on the function of other *Myc* genes in neural crest development, or about any developmental role of zebrafish *Myc* genes.

**Principal Findings:**

We isolated the zebrafish *mych* (*myc homologue*) gene. Knockdown of *mych* leads to severe defects in craniofacial development and in certain other tissues including the eye. These phenotypes appear to be caused by cell death in the neural crest and in the eye field in the anterior brain.

**Significance:**

Mych is a novel factor required for neural crest cell survival in zebrafish.

## Introduction

*Myc* genes function in cellular proliferation by regulating cell cycle progression, apoptosis, and cell transformation. Myc factors are thought of as regulators of gene transcription that activate or repress multiple target genes [1], [2], [3], [4], [5]. Myc proteins have two recognized functional domains, the N-terminal domain (NTD) and the C-terminal domain (CTD). The CTD contains a basic helix-loop-helix leucine zipper (bHLH-LZ) motif that is necessary for target DNA binding and regulation of gene expression. The Myc NTD contributes to the control of transcriptional activation or repression of downstream target genes [6], [7], [8], [9]. While the role of the Myc family in cell proliferation and cancer has received wide attention, comparatively less is known about its developmental functions.

Neural crest cells originate at the edge of the neural plate and at the dorsal aspect of the neural tube, and migrate to many locations where they differentiate into a great variety of cell types [10]. In *Xenopus, c-Myc* is expressed in early premigratory neural crest cells, and inhibition of its expression results in a loss of neural crest precursor cells and their derivatives [11]. The function of c-Myc in the neural crest involves *Id3*, a gene likewise expressed in neural crest precursors [12]. In the mouse, conditional inactivation of *c-myc* using *Wnt1-Cre* for targeted inactivation, led to skull, middle ear, and coat pigmentation defects [13]. These findings implicate *c-Myc* in the regulation of neural crest formation in the mouse. In zebrafish, *Myc* genes have been cloned and their expression has been reported [14], [15]. Here we present isolation of a novel member of the family, *mych* (for nomenclature see Zebrafish Information Network: http://zfin.org), and report its expression pattern and a functional analysis using morpholino-based depletion. *Mych* knockdown embryos experience excess apoptotic cell death in the neural crest population and in anterior brain. Subsequently, pharyngeal arches failed to develop properly and the eyes were smaller than normal and lacked laminar organization. Thus, *mych* function is required in neural crest and eye cell survival and development.

## Results

### Isolation of full-length *mych* cDNA and sequence analysis of Mych protein

**Figure 1:**
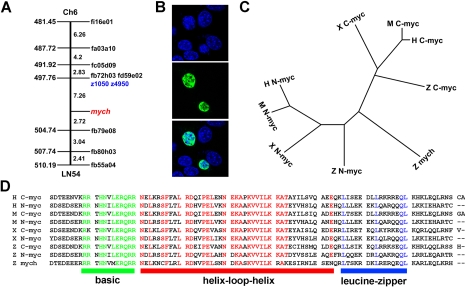
Characterization of *mych* and comparison of vertebrates Myc proteins. A. Radiation hybrid mapping determined the location of *mych* on chromosome 6. B. Nuclear localization of Flag-Mych protein (green) in NIH3T3 cells; nuclei were stained with DAPI (blue). C, D. Evolutionary comparisons. H, human; M, mouse; X, *Xenopus;* Z, zebrafish. C. Phylogenic tree of Myc family proteins. D. Amino acid sequence alignments of the C-terminal regions containing highly conserved basic helix-loop-helix domains. Amino acid identities are shown in color, with the basic, helix-loop-helix, and leucine-zipper domains marked in green, red, and blue, respectively. The GenBank Accession Number for *mych* is EU232118.

A partial *mych* cDNA was identified previously in an in situ hybridization-based gene expression screen [16]. We cloned full-length *mych* containing 1,975 base pairs (bp) using the 5′- RACE method. The cDNA predicts a 360-amino acid protein that contains a bHLH-LZ domain with high sequence identity with the CTD of vertebrate N-myc and C-myc proteins (57%–72%) (Fig. 1D). However there is only 39–57% identity in the NTD of Mych as compared to other Myc family proteins, and the amino acid sequences of the entire proteins are only 36–38% identical. A phylogenic tree of the Myc family based on the CTD shows that Mych is located between the c-Myc and N-Myc clusters, closer to N-Myc (Fig. 1C). The *mych* gene is located on chromosome 6 according to the Sanger genome center, version Zv7 (http://www.ensembl.org/Danio_rerio/index.html; Zv7 Scaffold638, contig BX649289.11), and confirmed by radiation hybrid mapping using the LN54 panel [17] (Fig. 1A). To address the intracellular localization of Mych protein, a Flag-tagged *mych* construct was transfected into NIH3T3 cells; the protein localized in the nucleus, as is generally true for Myc family proteins (Fig. 1B).

### Dynamic and specific expression of *mych* during early development

**Figure 2:**
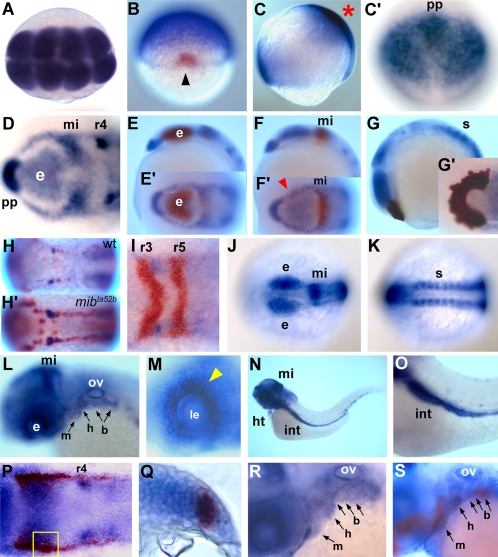
Detection of *mych* transcripts by in situ hybridization. A. Dorsal view of 8-cell stage embryo. B. Double staining of *mych* (blue) and *gsc* (red) at the shield stage; arrowhead points to the shield. C-C'. Lateral (C) and anterior dorsal (C') views of 80% epiboly stage. Red asterisk points to the anterior-dorsal area shown in C'. D–I. 3-somite stage embryos. D. Dynamic expression of *mych* in the anterior brain region. E-E'. Double staining with *mab21l2* (red) as a marker for eye and midbrain. F-F'. Double staining with *dmbx1a* (red) as marker for eye field and midbrain; red arrowhead indicates eye field. G,G'. Co-expression with *ctsl1b* (red), marking the prechordal plate. H-H'. Dorsal view of *HuC*-positive neuronal cells (red) and *mych* staining (blue) in wild type (H) and the *mib^ta52b^* mutant (H'). I. Rhombomeres 3 and 5 are marked by *egr2b* (red), while *mych* stains the anterior part of rhombomere 4. J–K. Dorsal views of 10-somite stage embryo showing *mych* expression in the eye and midbrain (J) and in trunk somites (K). L. Lateral view of *mych* expression in the brain and pharyngeal system at 24hpf; mandibular (m), hyoid (h), and branchial (b) arches are indicated. M. Expression of *mych* in the eye at the 36hpf. Arrowhead points to presumptive photoreceptor cell layer. N. *mych* expression in the heart and intestine (int; magnified in O) at 72hpf. P–Q. Co-expression with *foxd3* (red) at the 4-somite stage. The yellow open square area in P is shown as a section in Q. R–S. Pharyngeal arch marker *dlx2a* (red in S) is co-expressed with *mych* at 32hpf. b, branchial arch; e, eye; h, hyoid arch; ht, heart; int, intestine; le, lens; m, mandibular arch; mi, midbrain; ov, otic vesicle; pp, prechordal plate; r, rhombomere; s, somite.

The expression of *mych* in developing zebrafish embryos was examined by whole mount in situ hybridization. Maternal transcripts are broadly expressed and appear to persist at least through blastula stages (Fig. 2A). After shield formation, *mych* expression is excluded from the dorsal marginal area but is retained in the future prechordal plate region (Fig. 2B). During gastrulation, *mych* transcripts are found in distinct dorsal and ventral expression domains in the embryo (Fig. 2C,C′). To interpret the early brain expression pattern of *mych*, we carried out double staining with eye, tectum, and prechordal plate markers, using *mab21l2*, *dmbx1a/mbx*, and *ctsl1b/hgg1* [18], [19], [20]. At the 3-somite stage, *mych* is expressed in the eye field (Fig. 2E,E′,F,F′), midbrain (Fig. 2F,F′), and prechordal plate (Fig. 2G,G′). The bilateral expression of *mych* in the hindbrain was analyzed by double staining with *egr2b/krox20,* a marker for rhombomeres 3 and 5. *Mych* is expressed in rhombomere 4 (Fig. 2I), and this expression is strongly increased in the neurogenic mutant *mib^ta52b^* [21], suggesting that *mych* transcripts are present in neuronal cells in rhombomere 4 (Fig. 2H). *Mych* expression in the presumptive hindbrain begins at 90% epiboly (data not shown), and thus the gene is a very early marker for this region. During mid-segmentation stages *mych* is mainly expressed in the eye, midbrain, and somites (Fig. 2J,K). At 24hpf, mandibular, hyoid, and branchial arch expression increases (Fig. 2L), and eye expression is detected primarily in the photoreceptor layer at 36hpf (Fig. 2M). At 72hpf, brain, heart and gut show strong *mych* expression (Fig. 2N,O). To test whether *mych* is expressed during neural crest development we carried out two-color in situ hybridization with early neural crest marker *foxd3* [22] and the pharyngeal arch marker *dlx2a* [23]. At the 4-somite stage, *foxd3* and *mych* are colocalized in the region of the premigratory neural crest (Fig. 2P,Q), and at 32 hpf colocalization with *dlx2a* was seen in the pharyngeal arches (Fig. 2R,S). These observations indicate a wide but differential expression pattern for *mych* during embryonic development in the zebrafish.

### *Mych* knockdown phenotypes

**Figure 3:**
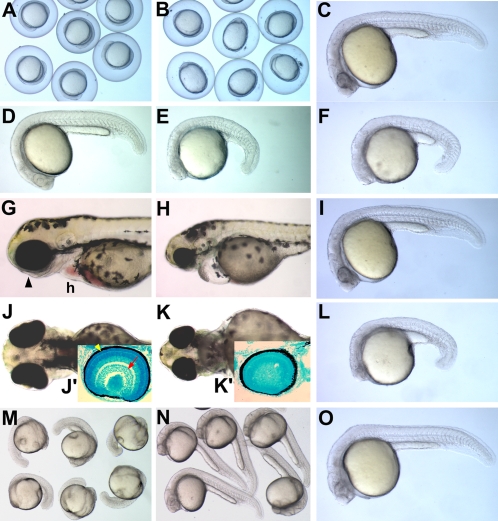
*Mych* morphant phenotypes. A–B. Group image of 3-somite stages. *Mych* MO-injected embryos (B) have reduced anterior and caudal regions compared to control MO-injected embryos (A). C–F. Lateral view of 24hpf MO-injected embryos; (C,D) Control, (E) *mych* UTR MO, (F) *mych* SP MO. G–H, J–K. Lateral (G–H) and ventral (J–K) views of control (G,J) and *mych* UTR MO injected embryos (H,K) at 72hpf. Arrowhead in G points to the eye. Methyl green stained sections of the eye are shown as insets (J',K').Yellow arrowhead in J' indicates ganglion cell layer, and red arrow indicates photoreceptor cell layer of control embryos; *mych* UTR MO-injected embryos show no retinal layering. F,I. Lateral view of 24hpf *mych* SP MO injected embryo (F), and embryo rescued by coinjection of *mych* mRNA (I). L,O. Embryo injected with *mych* UTR MO (2ng) plus *mych* SP MO (2.5ng) at 24hpf (L), and rescued embryo after coinjection of *mych* mRNA (O). M–N. The phenotype of *mych* UTR MO-injected embryos (M) was rescued by *mych* mRNA (N), as seen at 24hpf. h, heart.

**Figure 4:**
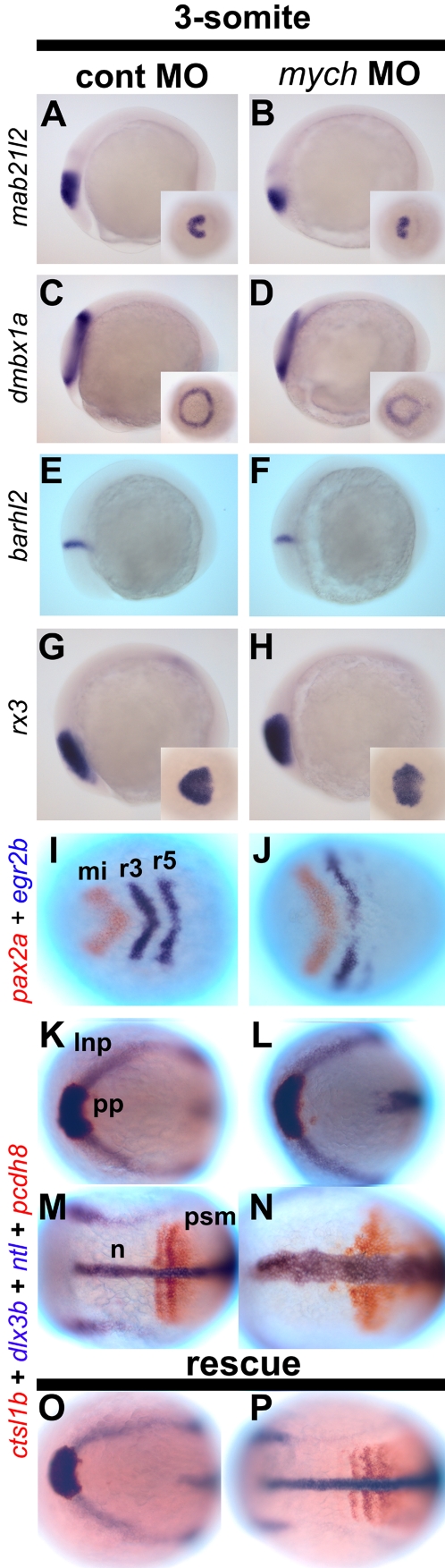
*Mych* MO affects multiple regions at the 3-somite stage. A–P. Lateral views (A–H) and dorsal views (I–P) of control MO-injected (A,C,E,G,I,K,M) and *mych* MO-injected embryos (B,D,F,H,J,L,N). *Mab21l2* (A–B') as eye anlage and midbrain marker; *dmbx1a* (C–D') as eye territory and tectum marker; *barhl2* (E,F) as diencephalon marker; *rx3* as eye field marker. (I–J) double label with *pax2a* (red) and *egr2b* (blue). (K–P). Embryos were examined using four probes, *ctsl1b* (red), *dlx3b* (blue), *ntl* (blue), and *pcdh8* (red). (O–P) *mych* MO-induced defects were rescued by co-injection with *mych* mRNA. lnp, lateral neural plate; mi, midbrain; n, notochord; pp, prechordal plate; r, rhombomere; psm, pre somitic mesoderm.

To study the function of *mych* in embryonic development, we used two morpholino oligonucleotides targeted against the 5′-untranslated region of the mRNA (UTR MO) and against the intron 1 splice donor site (SP MO). To determine MOs specificity, UTR MO was coinjected with mRNA for a fusion protein of Mych and GFP, showing a loss of GFP expression (Figure S1A,B). The SP MO specificity is examined by RT-PCR, using two sets of primers flanking the intron; the results show effective suppression of splicing (Figure S1C,D,E). *Mych* UTR MO injected embryos showed widening of the future trunk region at the 3-somite stage (75%, n = 109) (Fig. 3B and Fig. 4L,N) compared to control MO-injected embryos (5%, n = 77) (Fig. 3A and Fig. 4K,M). At 24hpf, UTR MO and SP MO-injected embryos (80%, n = 72) had a small head and reduced trunk (Fig. 3C–F). To confirm that the two MOs affect the same process we injected a combination of half-maximal doses of each MO into the embryo and observed a similar phenotype (Fig. 3L) (85%, n = 88). The phenotypes elicited by each MO and by the combination of MOs were rescued by coinjection of *mych* mRNA coinjection (Fig. 3I: 73% rescued, n = 65; Fig. 3M,N: 85%, n = 94; Fig. 3O: 70%, n = 68). Head and eye defects were also seen at later stages (Fig. 3G–H,J–K), and layering of the retina failed to develop normally in the UTR MO-injected embryos at 48hpf (Fig. 3J′,K′).

### Suppression of *mych* affects expression of anterior brain markers

**Figure 5:**
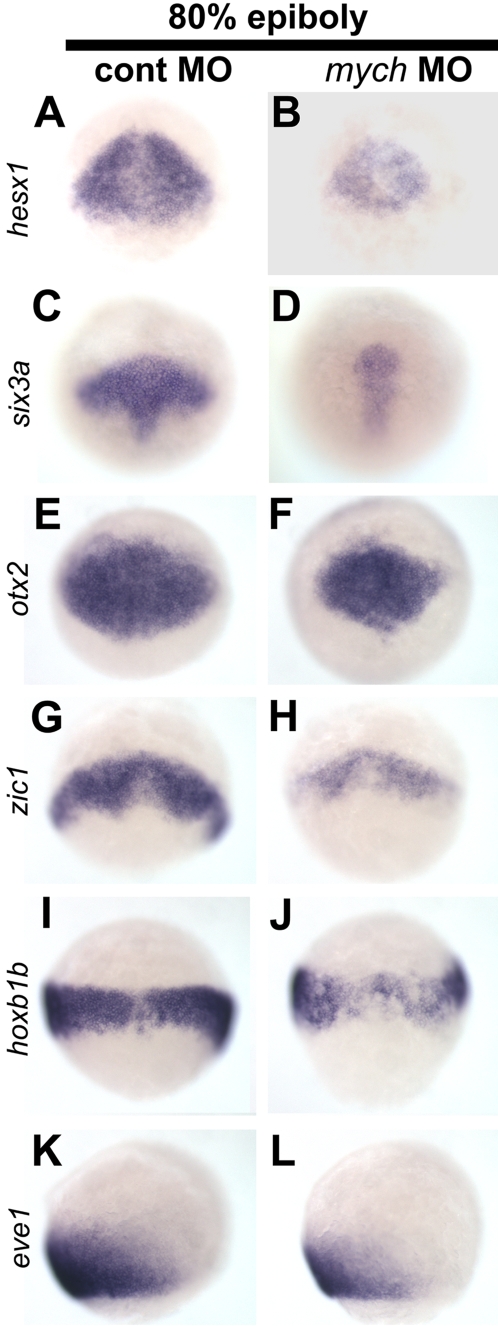
Reduced expression of early anterior brain markers after *mych* MO injection. A–J. Dorsal views (A–J) and lateral views (K–L) of control MO-injected (A,C,E,G,I,K) and *mych* UTR MO-injected embryos (B,D,F,H,J,L) at the 80% epiboly stage. The probes are indicated.

To further analyze the *mych* UTR MO phenotype we carried out in situ hybridization using early brain markers. During gastrulation the expression of *hesx1/anf, six3a, otx2*, and *zic1*/*opl* were dramatically reduced in intensity and in the size of their expression domains (Fig. 5A–H). The regions marked by these genes contribute to the specification of the eye territory and the telencephalon [24]. By comparison, the reduction in the expression of *hoxb1b* in the posterior hindbrain and *eve1* in the future trunk-tail were only slightly reduced in *mych* MO-injected embryos (Fig. 5I–L). Reduced anterior development in *mych* MO-injected embryos was also seen at the 3-somite stage. *Mab21l2* and *dmbx1a*, marking the eye and midbrain anlagen, were reduced in size and expression level in *mych* MO-injected embryos (Fig. 4A–D), while the eye-specific gene *rx3* showed a size but not intensity reduction (Fig. 4G,H). In contrast, the diencephalon marker *barhl2* was not altered by *mych* MO injection (Fig. 4E,F).

In addition to genes that mark the anterior neural plate we also tested posterior brain and trunk markers after injection of *mych* MO. Double staining with *pax2a* and *egr2b* in the mid- and hindbrain indicates that these areas were not greatly affected by *mych* MO, although *egr2b* expression in rhombomere 5 is reduced (Fig. 4I,J). Further, these markers suggest a widening of the axis, as also seen in unstained embryos (Fig. 3B). Such widening was further illustrated by using probes that label the polster (future hatching gland; *ctsl1b),* neural plate boundary and placodal anlage *(dlx3b*), the notochord (*ntl*), and early somites (*pcdh8/papc*). All of these markers were expressed in approximately normal patterns in the experimental embryos except that a widened axis was seen in 82% (n = 89) of *mych* MO-injected embryos, whereas 92% (n = 45) of control MO-injected embryos were normal (Fig. 4K–N). This axis abnormality was fully rescued by coinjection of *mych* mRNA (85% normal, n = 80) (Fig. 4O,P).

### Early neural crest specification requires Mych function

**Figure 6:**
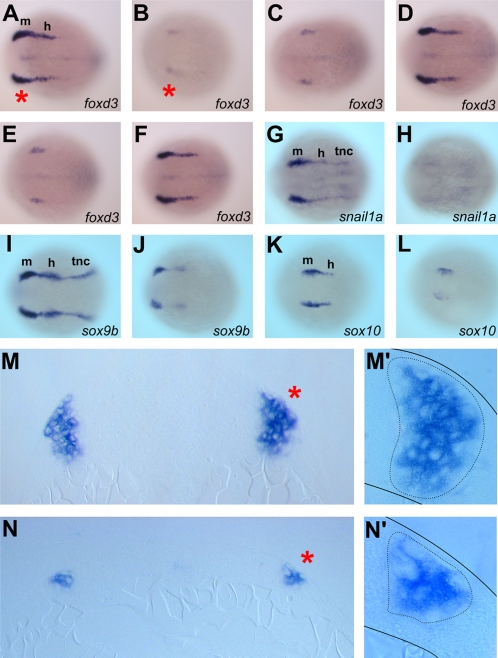
*mych* MO impairs early neural crest induction. A–F. Dorsal view of *foxd3* expression at 4-somite stage embryos. Embryos injected with 4ng of *mych* UTR MO (B), 5ng of *mych* SP MO (C), or 2ng of *mych* UTR MO (B) plus 2.5ng of *mych* SP MO (C) showed reduced *foxd3* expression. This phenotype was rescued by *mych* mRNA coinjection (D,F). G–L. Dorsal view of control MO-injected (G,I,K) and *mych* UTR MO injected embryos (H,J,L) hybridized to *snail1a* (G,H), *sox9b* (I,J), and *sox10* (K,L). M–N. Sections of the *foxd3* expression region of cont MO (M,M') and *mych* UTR MO (N,N') injected embryos. Red asterisks in A and B indicate the location of transverse sections, and asterisks in M and N indicate the regions magnified in M', N'. h; hindbrain neural crest, m; midbrain neural crest, tnc; trunk neural crest.

*Mych* is expressed in the mid- and hindbrain at early neural plate stages including the preplacodal regions (Fig. 2), and specifically in the early neural crest as seen by overlapping staining with the crest marker *foxd3* (Fig. 2P,Q). This pattern suggests a possible function in neural crest specification which proceeds at this stage at the junction between the neural and non-neural ectoderm [25]. We tested the effect of injection of *mych* MOs on the expression of early neural crest marker *foxd3* (Fig. 6A–F). The *foxd3* expression is dramatically reduced by *mych* UTR MO (Fig. 6B), SP MO (Fig. 6 C), and combination of half-maximal dose of both MOs (Fig. 6E). We also tested several early neural crest markers such as *snail1a, sox9b,* and *sox10* found them dramatically reduced in the midbrain region and completely lost in the hindbrain and trunk neural crest regions (Fig. 6G–L). To visualize the cellular context of the reduction in the expression of these genes we sectioned *foxd3*-stained embryos (Fig. 6M,N). The *foxd3* positive cells in the *mych* MO embryo contain a reduced number of neural crest cells and a reduced thickness of the region at the neural plate-to-epidermis boundary (Fig. 6M′,N′). We suggest that the inhibition of Mych expression led to a loss of neural crest precursors in the embryo, as supported by experiments below.

### *Mych* knockdown disrupts pharyngeal arch development

**Figure 7:**
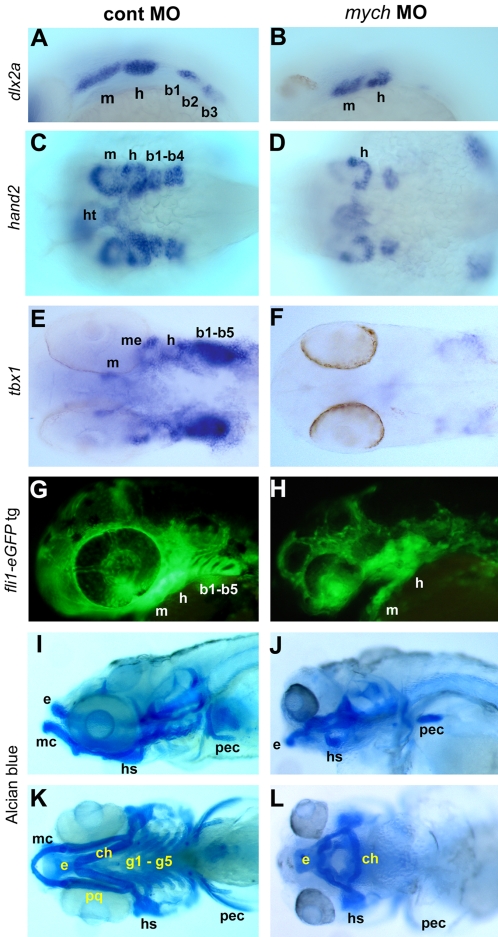
Multiple defects in pharyngeal arch development caused by *mych* MO. Control MO (A,C,E,G,I,K) and *mych* UTR MO-injected embryos (B,D,F,H,J,L). A–B. Lateral view of *dlx2a* expression in *mych* UTR MO-injected embryos at 26hpf (B). C–D. Ventral view of expression of *hand2* at 36hpf. E–F. Ventral view of *tbx1* expression at 45hpf. G–H. Lateral view of *fli1-eGFP* transgenic line at 40hpf. I–L. Lateral (I,J) and ventral (K,L) views of Alcian blue stained day 5 control (I,K) and *mych* UTR MO-injected embryos (J,L). ch, ceratohyal, b, branchial arch; e, ethmoid plate; g, gill arches; h, hyoid arch; hs, hyosymplectic; ht, heart; m, mandibular arch; mc, Meckel's cartilage; me, mesoderm; pec, pectoral fin; pq, palatoquadrate.

At 24hpf, *mych* is strongly expressed in the developing pharyngeal arches and maintained in this area for at least two days of subsequent development (Fig. 2). We observed effects of *mych* depletion on pharyngeal development by several approaches. Whole mount in situ hybridization at 26hpf showed that *dlx2a* and *tbx1* expression was lost in the branchial arches, while defects in the mandibular and hyoid arches were comparatively mild (Fig. 7A and B, and data not shown). At 36hpf, *hand2/dhand* expression in the branchial arches was likewise inhibited by *mych* MO (Fig. 7C,D). Mesodermal and endodermal development was likewise strongly inhibited after *mych* MO injection, as visualized by *tbx1* [26] at 45hpf (Fig. 7E,F). We also found a loss of *myod* expression in MO-injected embryos, indicating a loss of pharyngeal muscle (data not shown). To test whether these defects lead to loss of arch tissue, we injected *mych* MO into the *fli1-eGFP* transgenic line in which the cranial neural crest is visualized by GFP fluorescence [27]. A loss of branchial arches was seen, whereas portions of the mandibular and hyoid arch structures were maintained (Fig. 7G,H). These early pharyngeal defects lead to the loss of most parts of the cranial cartilages while most of the neurocranium was maintained, as seen by Alcian blue staining at day 5 (Fig. 7I–L). These data indicate that Mych is required for pharyngeal arch development in the zebrafish embryo.

### Loss of *mych* leads to increased cell death in the early neural plate

Myc family genes have been implicated in the regulation of cell proliferation. Therefore we tested whether inhibition of *mych* expression decreases cell division in the embryo. Using Phospho-Histone H3 antibody to identify proliferating cells we found no substantial difference between control and *mych* MO-injected embryos (data not shown).

**Figure 8:**
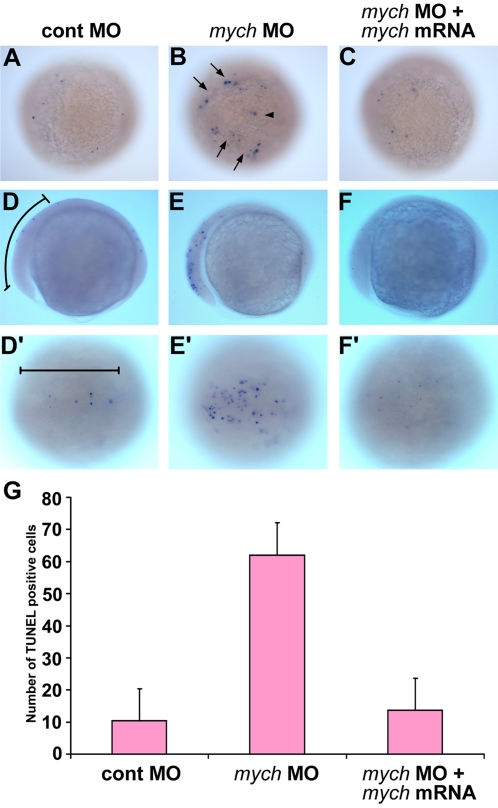
*Mych* depletion results in apoptosis in the early neural plate. A–F'. Detection of cell death by TUNEL assay. Lateral views (D,E,F) and dorsal views (A,B,C,D',E',F') of control MO (A,D,D'), *mych* UTR MO (B,E,E'), and rescued embryos that received *mych* UTR MO and mRNA (C,F,F') at the bud (A–C) and 3-somite stage (D–F'). Arrows in B point out TUNEL positive cells at the lateral edge of the neural plate. An arrowhead in B indicates TUNEL positive cells in the neural plate. Bracket in D and D' indicates the anterior neural plate region in which TUNEL-positive cells were counted. The results are shown in G. Average numbers of positive cells per embryo were obtained by counting 20 embryos in each group. For both comparisons p<0.01: Cont MO vs. *mych* MO: p = 5.27e-14; *mych* MO vs. rescue: p = 4.87e-17.

As the phenotypes resulting from *mych* depletion might also be caused by cell death, we tested for cell death at different times during gastrulation to segmentation stages. The earliest cell death was observed at about the 80% epiboly stage in the anterior dorsal region, with a moderate increase in the number of TUNEL-positive cells (data not shown). At bud stage, *mych* MO-injected embryos showed highly increased numbers of apoptotic cells, especially at the lateral edge of the neural plate and in the brain (65%, n = 67), as compared to control MO injected embryos, 95% of which showed very low levels of TUNEL-positive cells (n = 45) (Fig. 8A,B). The cell death phenotype was rescued by *mych* mRNA (80% normal, n = 76) (Fig. 8C). In the neural plate of such embryos at the 3-somite stage, a dramatic increase in TUNEL-positive cells was observed in 85% (n = 45) of *mych* MO-injected embryos (Fig. 8E,E′), as compared to 1% (n = 34) in control MO-injected embryos (Fig. 8D,D′). Again this phenotype was rescued by injection of *mych* mRNA in 80% (n = 65) of the embryos (Fig. 8F,F′). For statistical analysis, we counted the number of TUNEL-positive cells in the anterior neural plate in 20 embryos for each injection condition; the changes are highly significant (Fig. 6G). These data indicate that *mcyh* is important for the survival of neural plate cells in early embryo development.

## Discussion

We have isolated the *mych* gene as a novel member of the Myc family in zebrafish, and have shown that it is involved in the survival of cells in the neural plate including the region from which the neural crest is derived. Zebrafish Myc family genes, including *c-myc, N-myc, L-myc* and *max* have been isolated previously and the distribution of their transcripts has been reported [14], [15]. The Mych protein is related to other Myc proteins, showing low sequence similarity in the N-terminal Myc domain (NTD), but high similarity in the bHLH-LZ domain. As other Myc proteins, Mych is localized in the nucleus and may therefore function as a transcriptional factor (Fig. 1). *Mych* mRNA is expressed maternally, is restricted to a dorsal and a ventral domain at gastrulation, and later shows a dynamic expression pattern with a high level of expression in certain regions of the anterior brain. For comparison we carried out in situ hybridization with zebrafish *c-myc* and its binding partner *max*. Both were present maternally and were later expressed in a very broad pattern (data not shown). In contrast, *nmyc1* expression was only zygotic, and its tissue distribution in the endoderm, retina, midbrain, hindbrain and branchial arches is similar to that of *mych* [15]. While the *mych* expression pattern at gastrulation suggests an involvement of this gene in dorsal-ventral patterning we found no substantial changes in the expression of *bmp2, chordin,* and *goosecoid* in *mych* MO-injected embryos (data not shown).

Inhibition of *mych* expression resulted in reduced size of the anterior brain without major changes in patterning (Figs. 4 and 5). At later stages we observed strongly reduced size of the head and eyes. These phenotypes may be due to the fact that *mych* knockdown greatly increases the level of cell death in the anterior neural plate (Fig. 8). While several tissues are affected in *mych* MO-injected embryos, neural crest differentiation seemed especially sensitive to the loss of Mych function. Early markers of neural crest differentiation were strongly reduced by *mych* knockdown (Fig. 6), and the development of branchial arches was severely disrupted, while the effect was less extensive in the mandibular and hyoid arches (Fig. 7). Neural crest cells arise at the border of neural and non-neural domains, and subsequently migrate to multiple target organs [10]. After migration into the pharyngeal arches, neural crest cells undergo condensation and chondrogenic differentiation to form the cartilage elements of the developing craniofacial skeleton. Expression of *mych* is maintained in these regions during their differentiation. A function for *mych* is supported by the observation that knockdown of its expression causes reduction of known regulatory genes in the branchial arches, such as *dlx2a*, and ensuing morphological malformations (Fig. 7). These effects may be a consequence of the increased apoptosis in *mych* MO-injected embryos, as disruption of branchial arch development in conjunction with apoptosis as a result of loss of Ap2 transcription factor has been observed previously in zebrafish [28], [29]. The specific involvement of Myc family genes in neural crest development has been studied in *Xenopus* and in the mouse. In *Xenopus*, *c-Myc* is involved in early neural crest specification [11], and conditional deletion of *c-Myc* in the mouse results in neural crest defects, including reduction of skull size and deficits in coat pigmentation and hearing [13]. In *Xenopus*, c-Myc appears to have a role in maintaining neural crest stem cells by acting through its target Id3 to prevent premature differentiation [11.12]. Zebrafish often contains additional members of gene families as compared to tetrapods, due to a genome duplication during evolution [30]. The resulting paralogs often have partly overlapping, partly distinct functions that together correspond to the functions of the single ortholog in other vertebrates. Therefore it is possible that in zebrafish *mych* takes on some of the functions carried out by *N-Myc* and *c-Myc* in *Xenopus* or the mouse. Such a model might explain the role of *mych* in neural crest development, although it is not clear whether the molecular function of *mych* in zebrafish is similar to that of *c-Myc* in *Xenopus*. In spite of this uncertainty, the requirement for *mych* function in zebrafish neural crest development supports the view that Myc family members are essential regulators of neural crest development in all vertebrates.

## Materials and Methods

### Fish strains

Wild type zebrafish strains AB* were maintained according to The zebrafish book: A Guide for the Laboratory Use of Zebrafish (*Danio rerio*) [31]. The *mib^ta52b^* mutant line was obtained from Ajay Chitnis, and homozygote *fli1-eGFP^y1^* transgenic line from Brant M. Weinstein.

### Isolation of full-length *mych* cDNA and RH mapping

The original partial clone 5144 [16] contains 1.6 kb, and was extended to 1.9 kb full-length *mych* cDNA using the SMART RACE cDNA Amplification Kit (ClonTech). Radiation hybrid mapping was done with the LN54 panel [17], using two primer sets: (1) Forward 5′-GCCGCAAGGAGGATCTGCGGACTT-3′, Reverse 5′-AGATACTAAC TCCAGCTGGTCCAC-3′; and (2) Forward 5′-TCGCCGACGTTTTCCGTCTACTTT-3′, Reverse: 5′-CAGTTGGAGAAAGTCTGTGTCCTC-3′. Amino acid sequence comparisons and phylogenic tree analysis were carried out with DNASIS MAX version 2.0 (MiraiBio, Hitachi software).

### RT-PCR analysis

To isolate to total RNA, 20 each of cont MO and *mych* SP MO injected embryos were collected and isolated using TRIzol reagent (Invitrogen). The first-strand cDNA synthesis was performed SuperScript III First-Strand System (Invitrogen). RT-PCR reactions accomplished using two following sets of primers: Forward F1-CTGGACTGCCACAC CGCGGCGCTCGCCTG/Reverse R1-GCTGGAGCGCCGCACCGTCACCACATC; Forward F2- GACAGCCAGGAGCAGATCGAATCCAC/Reverse R2-GCACGCTGCT GCTCCCGCCGGCTGTCCTC. The amplified genomic DNA were confirmed by sequencing analysis.

### Whole mount in situ hybridization

Whole mount *in situ* hybridization and two-color *in situ* hybridization performed as previously described [32]. Both digoxigenin- and fluorescein-labeled antisense RNA probes were generated using an RNA labeling kit (Roche).

### *mych* MO and rescue mRNA injection

Antisense oligonucleotide Morpholinos (MO) were designed and obtained from Gene Tools, LLC. The *mych* 5′untranslated MO (UTR MO) does not contain AUG translation start site sequences. The sequence of the UTR MO is 5′- ACTGTGGTGATAAAAGT AGACGGAA-3′, The *mych* splicing MO was designed splicing donor region of intron between exon 1 and exon 2 (Figure S1 C). The sequence of the SP MO is 5′-GCAAAAGA CTCACCAGAATCGCTAG-3′, the control MO is 5′-CCTCTTACCTCAGTTACAATT TATA-3′. In all experiments 10ng of Control MO, 4ng of *mych* UTR MO, and 5ng of SP MO were injected into one-cell stage embryos. Full-length *mych* mRNA was subcloned into pCS2^+^ or pCS2^+^-eGFP1 vectors and synthesized by mMessagemMachine SP6 Kit (Ambion), and 30 pg mRNA per embryo were injected in rescue experiments.

### TUNEL assay and immunocytochemistry

TUNEL assay was performed as described previously [32]. The localization of Mych protein was determined after transfection of flag-tagged *mych* DNA into NIH3T3 cells using FuGENE 6 tranfection reagent (Roche). We used 1∶1000 dilution of the mouse mono clonal anti-flag antibody (Sigma), and 1∶2000 dilution of mouse Alexa 488 (Invitrogen) as a secondary antibody. DAPI was used to stain nuclei. The images were scanned in a Zeiss LSM 510 confocal microscope.

### Alcian blue and Methyl Green staining

Pharyngeal cartilage staining was carried out as previously described [32] using Alcian blue (Sigma). The 0.5% methyl green (Sigma) solution was prepared in 0.1 M sodium acetate buffer (pH 4.2). After 5 minute staining, samples were rinsed in water and dehydrated in 95% ethanol.

### Histology

For sections, dehydrated embryos were embedded in JB-4 plastic resin (Polyscience Inc.), and 7 µm sections were obtained using a Leica RM2165 microtome. The two-color double staining sample was embedded with 10% gelatin (Electron Microscopy Sciences) in 1XPBS and section was performed using a Vibratome 3000 (Ted Pella Inc.) with 10 µm thickness.

## Supporting Information

Figure S1 Mych MO specificity. A–B. Mych:GFP signal detection at the bud stage after injection with (B) or without (A) mych UTR MO. C. Schematic drawing of mych SP MO design and two different sets of RT-PCR primers. D–E. RT-PCR shows that the SP MO eliminates the normal mature mRNA band. Embryos were collected at the 3-somite stage.
(8.19 MB TIF)
