# Supplementary figures and images for: The Mych Gene Is Required for Neural Crest Survival during Zebrafish Development
Source: PLoS One. 2008 Apr 30;3(4):e2029. doi: 10.1371/journal.pone.0002029 (PMC2323570; doi:10.1371/journal.pone.0002029)

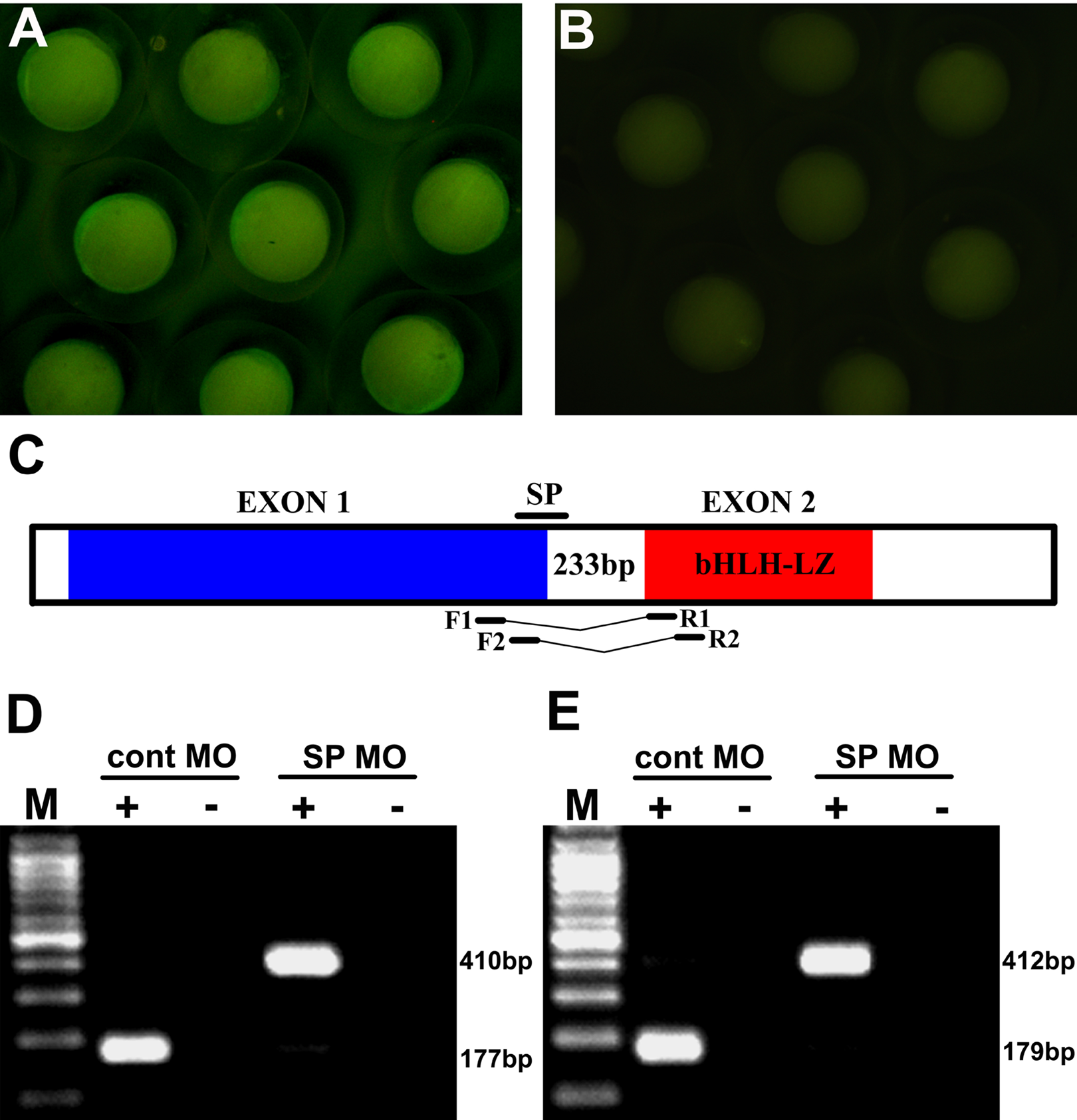

Supplement: Figure S1 — Mych MO specificity. A–B. Mych:GFP signal detection at the bud stage after injection with (B) or without (A) mych UTR MO. C. Schematic drawing of mych SP MO design and two different sets of RT-PCR primers. D–E. RT-PCR shows that the SP MO eliminates the normal mature mRNA band. Embryos were collected at the 3-somite stage. (8.19 MB TIF) [file pone.0002029.s001.tif]
